# 

**Published:** 1867-12

**Authors:** 


### CONTENTS.

| | PAGE |
|---|---|
| Twenty-first Announcement of the Medical Dept. Iowa State University | 1 |
| Proceedings of the Iowa State Medical Society | 12 |
| New Operation upon the shaft of Long Bones, by which Elongation as well as Straightening may be Secured; by Prof. J. C. HUGHES, M. D | 23 |
| Editorial — Iowa Medical Journal | 26 |
| Our Medical College | 2[illegible] |
| M'Gugin Monument | 27 |
| Our Advertising Friends | 28 |

